# Supplementary material for: Is Türkiye’s on-the-job training program sustainable? A qualitative evaluation from the firms’ perspective
Source: PLoS One. 2026 Feb 23;21(2):e0341254. doi: 10.1371/journal.pone.0341254 (PMC12928438; doi:10.1371/journal.pone.0341254)
Supplement: Attachment — Submitted filename: Review of PLOS ONE journal paper.docx [file pone.0341254.s002.docx]

# Review of PLOS ONE journal paper: Is Türkiye's on-the-job training (OJT) program sustainable? A qualitative evaluation from the firm's perspective.

Reviewer's comments:

**Summary**: The structure of the subject matter is presented in a coherent manner, effectively highlighting the gaps in evidence and the rationale for the current study.

I believe results shared in this paper are relevant and could inform improvements to the implementation process of the OJT program.

However, there are a few areas that need to be addressed to further improve the quality of evidence presented in this paper.

**Introduction**: Decision to only explore managers' perceptions of the OJT program: I believe the experiences of both trainers (managers responsible for OJT) and recipients (employees receiving OJT) would vary significantly. Interviewing both managers and employees, in my opinion, would provide a more accurate assessment of the program's effectiveness and contribution. Relying solely on managers' perspectives may result in a biased, one-sided view. However, a justification of why the focus is on the employer's perception alone may be sufficient to address this concern.

**Line 79**: While your research questions are generally relevant for a qualitative study, they could be refined to enhance clarity, depth, and alignment with a qualitative inquiry. As it is, with the current "**what**" questions, it is more tethered to a quantitative study. See the below suggestions.

- How do firms recruit participants for their programs, and what criteria and methods do they use?
- What challenges do firms face in recruiting participants for their programs?
- How do firms experience and perceive the training, audit and implementation of their programs?
- What are the firms’ views on the main outputs and impacts of their programs in terms of satisfaction, suggestions, and employment?

**Line 101**: For me, I don't think it is relevant to add the last paragraph on how the paper is sectioned.

**Literature review**: thoroughly captures up-to-date evidence and what is out there and how it works in this context, with well supported arguments.

**Materials and methods**: methodological approach appears to be very thorough and rigorous, with appropriate sampling and interviewing methods. I was particularly impressed with the use of NVivo software for data analysis - which I believe is not only great at organising and managing the data effectively, but it improves the quality of data/code queries and visualisation of insights gleaned. And this was evident in the outcomes/results presented.

The **data analysis** section lacks sufficient detail regarding the generation of codes and the determination of final codes and subcodes. Currently, it is overly generic and cannot be replicated. Despite potential word count constraints, it is important to report the process clearly and accurately. The coding process needs to be explained comprehensively to show how codes were derived from the text or how patterns and overarching ideas emerged from combined codes, leading to the identification of themes. Additionally, it should be clarified whether you reviewed the data to confirm that emerging themes accurately represented the codes and original data, and if refinements were needed. Providing these details is crucial to demonstrate the rigour of the analytical process and support the validity of the findings.

**Results**: this section was well-structured and clearly presented.

**Table 3**: While I appreciate the idea of using a cluster analysis in inferring the benefits of the programme, I wasn't convinced it was appropriate. It is true that cluster analysis groups data based on similarities, often used to identify patterns across datasets – which could apply to qualitative data. However, its utility in interpretive/narrative studies is often limited. This limitation arises because cluster analysis may overlook the intricate, individualised narratives that this study aims to capture. With studies exploring the perceptions of people it is important to maintain the richness and integrity of people's personal experiences, hence why relying on thematic coding and direct interpretation is often better suited. So, to quantify these interpretations, for me, takes away from those experiences. But this is just my opinion. I'd be keen to see what other reviewers think of this.

So, this can be reconsidered or captured as a limitation, which I noticed your paper is missing a limitation section.
