# Supplementary material for: Is Türkiye’s on-the-job training program sustainable? A qualitative evaluation from the firms’ perspective
Source: PLoS One. 2026 Feb 23;21(2):e0341254. doi: 10.1371/journal.pone.0341254 (PMC12928438; doi:10.1371/journal.pone.0341254)
Supplement: Attachment — Submitted filename: response to reviewers OJT.docx [file pone.0341254.s004.docx]

Dear Reviewers,

We sincerely appreciate your thoughtful comments and constructive feedback, which have significantly enriched our manuscript. In response, we carefully considered each point raised and revised the manuscript accordingly.

All modifications made following the initial submission are documented in detail in the tracked-changes version of the manuscript. In this version, we have included comment boxes throughout the text specifying exactly which reviewer’s feedback prompted each revision.

Below, we provide a summary of the changes made and our responses to your comments.

**RESPONSE TO REVIEWER #1**

**Reviewer 1 Comment 1:**

**The decision to only explore managers’ perceptions may provide a one-sided view. Interviewing both managers and employees would improve accuracy. A justification for focusing only on employers may suffice.**

Thank you for this insightful comment. In the revised Introduction, we have now provided a clear rationale for focusing exclusively on employer perspectives. We emphasized that firms are the primary agents responsible for implementing OJT practices, making hiring decisions, and evaluating performance—thus offering critical insights into the program's operational effectiveness. While we acknowledge the importance of employee perspectives, we clarify that this will be explored in a planned second-phase study. We have interpreted the data obtained from employers within the framework of human capital, labor market segmentation and implemenatation gap approximations.

**Reviewer 1 Comment 2:**

**Research questions are too descriptive ("what") and suggest a quantitative design. Consider more interpretive phrasing.**

We have reworded all four research questions to reflect an exploratory qualitative orientation. For example, the first question now reads: *“How do firms recruit participants for the OJT program, and what criteria and methods do they use?”* The changes aim to align the inquiry more closely with thematic and interpretive analysis.

We have revised the structure of the Results section to ensure that each thematic finding clearly corresponds to a specific research question. We believe that this alignment enhances the narrative coherence and analytical depth of the manuscript.

**Reviewer 1 Comment 3:**

**Remove the last paragraph that outlines how the paper is structured. (For me, I don't think it is relevant to add the last paragraph on how the paper is sectioned.)**

As suggested, the paragraph outlining the manuscript’s structure at the end of the Introduction has been removed to improve flow.

**Reviewer 1 Comment 4:**

**Literature review thoroughly captures up-to-date evidence and what is out there and how it works in this context, with well supported arguments.**

Thank you for your feedback. In response to Reviewer 3’s criticisms regarding the literature review, we have engaged in a more theoretical discussion without departing from the content you appreciated. We also aimed for a more systematic presentation by introducing subheadings. Compared to the previous version, we have better reflected the explanatory power of the theoretical discussion and the selected theories in relation to the research problem. We hope that you will find this version more satisfying.

**Reviewer 1 Comment 5:**

**The coding and thematic analysis process is too generic. Please clarify how codes were developed and how themes emerged. (The data analysis section lacks sufficient detail regarding the generation of codes and the determination of final codes and subcodes. Currently, it is overly generic and cannot be replicated. Despite potential word count constraints, it is important to report the process clearly and accurately. The coding process needs to be explained comprehensively to show how codes were derived from the text or how patterns and overarching ideas emerged from combined codes, leading to the identification of themes. Additionally, it should be clarified whether you reviewed the data to confirm that emerging themes accurately represented the codes and original data, and if refinements were needed. Providing these details is crucial to demonstrate the rigour of the analytical process and support the validity of the findings.)**

We have substantially revised the materials and methods section. The revised text explains our hybrid approach combining open coding and grounded theory, codebook development, axial theme construction, and validation through inter-coder reliability and NVivo analytics. A clear description of memoing, theoretical saturation, and theme refinement is now provided. We also made an addition to the conclusion section.

**Reviewer 1 Comment 6:**

**Table 3’s use of cluster analysis seems inappropriate for a narrative-based study. Consider removing or reframing. While I appreciate the idea of using a cluster analysis in inferring the benefits of the programme, I wasn't convinced it was appropriate. It is true that cluster analysis groups data based on similarities, often used to identify patterns across datasets – which could apply to qualitative data. However, its utility in interpretive/narrative studies is often limited. This limitation arises because cluster analysis may overlook the intricate, individualised narratives that this study aims to capture. With studies exploring the perceptions of people it is important to maintain the richness and integrity of people's personal experiences, hence why relying on thematic coding and direct interpretation is often better suited. So, to quantify these interpretations, for me, takes away from those experiences. But this is just my opinion. I'd be keen to see what other reviewers think of this.**

We greatly appreciate your insights on the methodological alignment between cluster analysis and narrative-based qualitative studies. In light of your comment, we have carefully reconsidered the appropriateness of including cluster analysis in this context. Rather than removing Table 3, we have now reframed its use in the Results section as a supplementary pattern-identification tool, while clearly stating its limitations. We also added a statement in the Discussion noting that such visualizations should not replace interpretive analysis and that future studies might reconsider their use. A limitation regarding this issue is now explicitly included in the new “Limitations” paragraph.

**RESPONSE TO REVIEWER #2**

**A pragmatically important paper indicating that Türkiye's on-the-job training (OJT) program although OJT holds significant potential for promoting sustainable employment, further improvements are necessary to enhance its impact.**

Thank you for your encouraging feedback, which motivates us to further improve our research

**RESPONSE TO REVIEWER #3**

**Reviewer 3 Comment 1:**

**The manuscript lacks a clearly articulated theoretical or conceptual framework.**

**Although the introduction and literature review touch on the relevance of ALMPs and OJT to Sustainable Development Goals, the manuscript lacks a clearly articulated theoretical or conceptual framework guiding the research. Explain your contribution to the literature (e.g., policy evaluation, firm-level engagement with state-sponsored programs, sustainability in active labor market tools) and situate your findings within relevant theoretical debates (e.g., labor economics, human capital theory, institutional theory).**

Thank you for your valuable comment. In the revised manuscript, we have strengthened our analysis by drawing on Human Capital Theory to explain how OJT programs aim to enhance employability by improving workers’ skills through firm-based training. Using Labor Market Segmentation Theory, we highlighted how the benefits of OJT may be unevenly distributed across sectors, occupations, and demographic groups, potentially reproducing existing labor market inequalities. We also employed the Implementation Gap Approach to interpret the disconnection between the policy’s stated objectives and its real-world outcomes. In response, we revised the Introduction section to clearly articulate the study’s conceptual framework. Additionally, we clarified our contribution to the literature in terms of regional implementation dynamics and the role of OJT in supporting SDG 8 (Decent Work and Economic Growth). We are grateful for this suggestion, which has helped us significantly improve the theoretical clarity and scholarly positioning of the manuscript.

**Reviewer 3 Comment 2:**

**The literature review is lengthy and somewhat unfocused. While it provides a broad overview of ALMPs, it could be improved by (a) grouping studies thematically (e.g., the impact of OJT on skills, firm-level perspectives, and gender inclusivity) and (b) highlighting gaps in the existing literature that this study directly addresses. Several citations appear descriptive rather than critically engaged.**

In response, we have fully revised the literature review section by thematically organizing it into 3 subsections: (1) *OJT and Skill Development*, (2) *Firm-Level Implementation and Experiences*, (3) *Gender, Inclusivity, and Sustainability.* This structure clarifies the flow of arguments, improves coherence, and helps highlight the specific gaps our study addresses. We also critically engaged with key references to move beyond descriptive summaries and to emphasize the study’s original contribution. Moreover, we have incorporated a clearer articulation of the study's contribution and the gap it fills into the Introduction section.

**Reviewer 3 Comment3:**

**While Antalya's tourism-dominant labor market is a compelling context, more justification is needed regarding why this province, despite its sectoral specificity, offers generalizable insights about the national OJT program. Are findings region-specific, or do they indicate systemic program features?**

In response, we revised the introduction and discussion sections to better explain the rationale for selecting Antalya as a case site. We emphasize the region’s sectoral diversity, its prominent role in implementing TEA’s OJT program, and its relevance for identifying systemic implementation challenges. We also clarify which findings are generalizable and which may reflect regional or sectoral specificities.

**Reviewer 3 Comment 4:**

**The research questions are listed clearly but lack integration into the narrative. Each question should link to relevant theoretical expectations or policy debates. Also, consider aligning your findings sections more explicitly with these questions.**

In the revised manuscript, we have refined the research questions to better reflect an interpretive qualitative approach and directly tied each question to the conceptual framework. This alignment has enhanced the narrative coherence and analytical depth of the manuscript, and we are grateful for the reviewer’s guidance in improving this aspect of our study.

To further align with the research questions, we also updated the Results section headings as follows:

**4.1. Recruitment Practices and Challenges** (corresponds to RQ1 & RQ2)

**4.2. Experiences with OJT Implementation** (corresponds to RQ3)

**4.3. Perceived Outcomes and Employment Transitions** (corresponds to RQ4)

**4.4. Criticisms and Suggestions for Improvement** (extends RQ4 into future policy and implementation critique)

**Reviewer 3 Comment5:**

**Clarify grounded theory vs. thematic analysis. Explain how NVivo was used. Using grounded theory and thematic analysis is ambiguous and could confuse readers. Please clarify whether the study adopts an inductive or hybrid coding approach and explain how grounded theory methods (e.g., memo writing, theoretical saturation) were used. Additionally, elaborate on how NVivo was applied beyond coding (e.g., query tools, clustering).**

We sincerely thank the reviewer for pointing out this important clarification. In response, we have revised the Data Analysis subsection (Section 3.4) to specify that our study employed a hybrid coding approach, integrating both inductive thematic analysis and grounded theory techniques such as memo writing, constant comparison, and theoretical saturation. We have also elaborated on the use of NVivo 14, detailing how it supported not only code management but also advanced features such as matrix queries, word frequency analysis, and cluster visualizations to enhance analytical depth and transparency. These additions have clarified our methodological rigor and analytic process, as suggested.

**Reviewer 3 Comment 6:**

**Justify sample size and sectoral diversity. The study uses purposive sampling but does not adequately justify the selection of 35 firms or how saturation was assessed. Discuss how sample size and diversity across sectors were sufficient to answer your research questions and whether regional sectoral biases might affect your results.**

In the revised manuscript, we have clarified our rationale for selecting 35 firms through purposive sampling by emphasizing sectoral representation, the strategic relevance of Antalya as a region with high participation in on-the-job training, and the diversity of firm types, including those operating in the service sector—primarily tourism—as well as in industry and agriculture. We also now explicitly discuss how thematic saturation was assessed through iterative coding and memo writing, following grounded theory procedures. Additionally, we acknowledge potential regional and sectoral limitations in the discussion, noting that findings may reflect Antalya’s tourism-driven context and encouraging comparative studies across different regions in future research.

**Reviewer 3 Comment 7:**

**Results section is overly descriptive and lacks quotations. The results section contains rich qualitative insights. However, it is weakened by an over-reliance on figures and tables without adequate textual interpretation. Ensure that each theme is analytically discussed and that quotations are integrated to illustrate key findings. Currently, the results are often read as descriptive summaries.**

In the revised Results section, we have substantially enriched the narrative by providing more analytical commentary under each theme and integrating direct participant quotations to illustrate key findings. We also revised subheadings to align closely with the research questions, ensuring clearer thematic coherence. While tables and figures remain to support transparency, the textual analysis has been significantly deepened to better reflect the qualitative richness of the data, as recommended*.*

**Reviewer 3 Comment 8:**

**Gender inequity is mentioned but under-theorized. The discussion rightly identifies that gender-based inclusion is lacking in the current OJT system. However, this is not adequately theorized or linked to broader debates in labor policy, equity, or intersectionality. This topic deserves more critical engagement and could serve as a distinctive contribution to the paper.**

In response, we have expanded the Discussion section to engage more critically with the issue of gender inclusion. We now link our findings to broader debates in labor policy, equity, and intersectionality, highlighting the structural and cultural barriers that limit women’s access to OJT opportunities. We also propose targeted policy mechanisms—such as proportional affirmative action or sector-specific gender incentives—to address this gap. We are grateful for this suggestion, which has helped us enhance the paper’s contribution to inclusive labor market discourse.

**Reviewer 3 Comment 9:**

**OJT's claim to contribute to sustainable development is repeated but under-analyzed. Sustainability (economic, social, or environmental) should be more clearly defined in the context of labor policy. Consider engaging with SDG 8 ("Decent Work and Economic Growth") more analytically, possibly contrasting policy intentions with implementation realities.**

We expanded our analysis by theorizing sustainability within the framework of SDG 8, and critically examined the gap between the OJT program’s policy rhetoric and its implementation, highlighting economic, social, and environmental dimensions of sustainability in labor policy. Necessary edits have been added to the literature and discussion section.

**Reviewer 3 Comment 10:**

**The manuscript would benefit from a thorough language edit for clarity, grammar, and flow. Some phrasing is awkward (e.g., "the general approach of firms is harmonious with TEA"), and specific technical terms (e.g., "Jaccard Coefficient" in clustering) are insufficiently explained for a broad audience.**

We have clarified the function of statistical measures such as the Jaccard Coefficient through inline notes and provided interpretive context for cluster analysis figures. While the raw qualitative data remain in Turkish and are anonymized to protect participant confidentiality, we have added illustrative (translated) excerpts throughout the results section. We have also ensured that all analyses are transparently explained for replication and interpretation. We have identified and removed this and similar issues throughout the manuscript.

**Reviewer 3 Comment 11:**

**While ethics approval is noted, compliance with PLOS ONE data availability standards must be ensured. Currently, stating that data are in Turkish and anonymized is insufficient—clarify the exact conditions for access and provide at least illustrative (translated) data excerpts if permitted.**

In response to your comment, we have ensured compliance with PLOS ONE's data availability standards. The underlying dataset has now been made publicly available and uploaded to the system as part of the manuscript revision.

**Reviewer 3 Comment 12:**

**The conclusion reiterates earlier points without offering strong final insights. Please summarize the key contributions, revisit your research questions, and outline more sharply the implications for policy and future research. The recommendations for TEA would benefit from prioritization and contextual grounding.**

We have significantly revised the Conclusion section to enhance its analytical depth and policy relevance. The revised version now (1) summarizes the main contributions of the study more clearly, (2) explicitly revisits each of the four research questions with reference to our key findings, and (3) articulates more sharply the implications for labor market policy, particularly regarding the design and implementation of the OJT program by TEA.

We also prioritized our policy recommendations based on frequency and intensity of employer concerns (e.g., pension coverage, program flexibility, gender inclusivity), and provided contextual explanations for each. Furthermore, the conclusion now includes a specific call for future research in different regional and sectoral settings to assess the generalizability of our findings.

Together with revisions to the theoretical framework, literature review, and methodological clarity, we believe these changes strengthen the manuscript’s overall contribution and align it more closely with the expectations of PLOS ONE's interdisciplinary readership.
