# Supplementary material for: Is Türkiye’s on-the-job training program sustainable? A qualitative evaluation from the firms’ perspective
Source: PLoS One. 2026 Feb 23;21(2):e0341254. doi: 10.1371/journal.pone.0341254 (PMC12928438; doi:10.1371/journal.pone.0341254)
Supplement: Attachment — Submitted filename: Response to reviewer revision 2.docx [file pone.0341254.s005.docx]

**Dear Reviewer and Editor,**

We sincerely appreciate your thoughtful comments and constructive feedback, which have greatly contributed to improving the quality of our manuscript. In response, we have carefully addressed each point raised and revised the manuscript accordingly. We are uploading the complete dataset as Supporting Information to ensure transparency and facilitate further verification of the study’s findings. All modifications have been clearly indicated in the tracked-changes version of the manuscript. Below, we provide a concise summary of the revisions made and our responses to your comments.

| **Reviewer No:3** | **Response** |
| --- | --- |
| 1. While the revised manuscript references SDGs and includes more policy context, it still lacks a compelling conceptual or theoretical framework. Terms such as “sustainability,” “inclusive labor markets,” or “institutional support” are used descriptively but not analytically. Anchoring the study more clearly in relevant theoretical perspectives, such as institutional theory, human capital theory, or the literature on public-private coordination in ALMPs, would deepen the academic contribution. | In the revised manuscript, we have strengthened the conceptual foundation of the study. While other frameworks, such as institutional theory, were also considered, we found that human capital theory, the implementation gap perspective, and labor market segmentation theory were more suitable for our research focus. Specifically, human capital theory provides a clearer lens for understanding skill formation and productivity outcomes; the implementation gap perspective helps identify discrepancies between policy design and practice; and labor market segmentation theory explains the structural inequalities that constrain the program’s inclusiveness. These approaches were therefore integrated into the analysis to more effectively interpret the dynamics of OJT programs.  Additionally, we have used key terms such as “sustainability” and “inclusive labor markets” in a more analytical and theory-informed manner, both in the literature review and in the discussion of empirical findings. These revisions aim to clarify the study’s theoretical orientation and enhance its overall academic contribution. |
| 2. Although your use of direct quotes has increased, many of the findings are still presented in a primarily descriptive manner. There is an opportunity to move beyond summarizing employer sentiments and instead interpret what these views reveal about structural limitations, power dynamics, or unintended consequences of policy design. For instance, your observations about firms’ preference for short-term labor subsidies could be linked to broader discussions on dependency effects or passive employer behavior in ALMPs. | We enriched the Results section with interpretive paragraphs addressing structural segmentation, institutional power dynamics, and unintended policy consequences. These additions move the findings beyond descriptive summaries by linking employer narratives to systemic mechanisms shaping OJT outcomes. A new closing paragraph now synthesizes these insights and leads into the Discussion section. |
| 3. The use of cluster analysis via NVivo remains insufficiently justified. If you are maintaining a qualitative paradigm, statistical clustering based on code co-occurrence should be used cautiously and interpreted transparently. Otherwise, it risks appearing as a procedural rather than conceptual contribution. If you choose to retain this technique, please clarify what analytical insight it generated that would not have emerged from thematic coding alone. | We thank the reviewer for this critical point regarding the justification of cluster analysis. In response, we have revised Section 3.2 to clarify the methodological role of this technique as an exploratory and complementary tool. The updated text now emphasizes how the analysis visually confirmed the coherence of key thematic clusters, such as *discipline–motivation* and *cohesion–personal care*. Furthermore, we have elaborated on how this technique provided a unique analytical insight by demonstrating that the conceptual links we identified were also grounded in the participants' frequently repeated phrases and specific linguistic patterns. This structural evidence reinforces our interpretive findings from a different analytical angle. To enhance transparency, a brief summary of the procedure and the resulting dendrogram have also been included. |
| 4. The rationale for choosing Antalya as the study site has been strengthened, but the question of generalizability remains open. It would be helpful to more explicitly acknowledge the regional specificity of your findings and reflect on how these might vary in other industrial contexts across Türkiye. | We added a new paragraph in the Discussion section explicitly addressing analytic generalization and contextual transferability. The revised text explains how findings from Antalya’s tourism-driven economy may apply to other service-oriented regions while outlining the limitations of transferring results to industrial or agricultural contexts. |
| 5. While you reference SDGs throughout the manuscript, the discussion of sustainability remains somewhat rhetorical. It would strengthen the paper to offer a more precise articulation of what constitutes “sustainability” in OJT, economic viability for firms, social inclusion, long-term employment outcomes, or institutional stability, and to assess how your findings align with or challenge this framework. | We strengthened the discussion of sustainability by adding a new paragraph in Discussion. It redefines sustainability as a multidimensional construct—economic, social, and institutional—links it explicitly to SDG 8, and ties the concept back to our empirical findings from Antalya, explaining both its current fragility and its potential for long-term transformation. |
| 6. The revised manuscript is more readable and coherent, but minor grammatical and stylistic issues persist. A final round of professional language editing is recommended to enhance the clarity and fluency of the text. | Minor grammatical and stylistic revisions have been made throughout the manuscript. Consistent terminology (e.g., “OJT programs”), uniform table titles, and improvements in sentence fluency and clarity have been carefully implemented. |
| 7. The data availability statement is improved, and the manuscript now includes more illustrative quotes. While this is appreciated, readers would benefit from a more detailed codebook or appendix summarizing your thematic structure (e.g., themes, sub-themes, sample codes) to support transparency and reproducibility further. | We would like to thank the reviewer for their insightful and constructive feedback. We appreciate their acknowledgment of the improved data availability statement and the inclusion of additional illustrative quotes. In response to the valuable suggestion for enhancing the transparency and reproducibility of our research, we have now supplied a comprehensive codebook, a thematic file detailing the hierarchy of our themes, categories, and codes, and the fully anonymized raw data as supplementary materials with this revision. We are confident that these additions will provide readers with a clear and thorough overview of our analytical process, directly addressing the reviewer's comments and further strengthening the manuscript. |
