# Supplementary material for: Is Türkiye’s on-the-job training program sustainable? A qualitative evaluation from the firms’ perspective
Source: PLoS One. 2026 Feb 23;21(2):e0341254. doi: 10.1371/journal.pone.0341254 (PMC12928438; doi:10.1371/journal.pone.0341254)
Supplement: Attachment — Submitted filename: Response to Reviewers revision 3.docx [file pone.0341254.s006.docx]

**Response to Reviewer**

We would like to thank the Academic Editor and the reviewer for their careful evaluation of our manuscript and for the constructive comments that helped improve its clarity and structure. All comments have been fully addressed, as detailed below.

**Reviewer**

**Comment 1:**

*The authors should use academic language avoiding personal pronouns (e.g., “we”).*

**Response:**

The manuscript has been thoroughly revised to remove all first-person plural pronouns (e.g., “we,” “our,” “us”) from the academic narration. An impersonal academic style has been adopted consistently throughout the text, while preserving the original meaning, analytical arguments, and methodological rigor of the study.

**Comment 2:**

*The research questions should be placed after the literature review. Before the research questions, a paragraph should be included to clarify the research gap addressed by the study.*

**Response:**
The Research Questions have been reorganized to follow the Literature Review, placed immediately after the paragraph at the end of Section 2.3 that identifies the research gap and outlines the contribution of the present study. This reorganization establishes a smooth and coherent transition from the literature review to the methodological section by explicitly linking the identified gaps to the research questions that guide the empirical analysis.
